# Bilateral Operation in the Female for Calculus

**Published:** 1854-12

**Authors:** David W. Yandell

**Affiliations:** Nashville, Tenn.


Bilateral Operation in the Female for Calculus.—By David W. Yandell, M. D., of Nashville, Tenn.—Sarah W——, of Christian county, Ky., aged 13 years, the subject of stone since early injancy, her mother thinks from birth, applied to me in July last for relief. I wished to defer an operation until the pleasant days of autumn, and so contented myself by suggesting to the girl's physician, Dr. Thomas, of Hopkinsville, the usual palliative and invigorating treatment.

Early in August, Dr. Thomas advised me that the sufferings of the patient were so severe, and her general health declining so rapidly, that he deemed an immediate removal of the stone necessary.

On the 24th of August, when I first visited Sarah, she was very thin, pale and feeble, considerably exhausted by frequent paroxysms of severe vesical pain, and the subject of constant fever. Her physical development, generally defective, was especially so in the genital organs. The external portion of the vaginal canal was very contracted—the hymen almost imperforate.

The next day, with 120 beats to the minute, the patient was secured for the operation. Chloroform was administered, the bladder explored, and a stone of large volume at once detected. I adopted the operative procedure conceived and first executed by Prof. Eve, of the Nashville University, in 1852. The incisions were freely made, dilatation was effected by the finger, and the forceps introduced and attempts made at extraction. These failed because of the size of the calculus. I withdrew the forceps, re-introduced the lithotome and enlarged the wound. Efforts for removal were again unsuccessful. I now endeavored to crush the stone—the force employed bent the smaller sized lithotomy forceps. These were laid aside, and with a larger instrument I succeeded in breaking the stone into two large and a number of smaller fragments.

As the operation now promised to be very tedious, the patient was allowed to recover her consciousness. The work was proceeded with—fragment after fragment, numbering in all nearly one hundred, and well nigh two tea-spoonsful of sandy particles were extracted. The bladder was thoroughly cleansed—the patient unbandaged, placed in bed, and a full opiate given. In five hours after the operation she urinated at will with almost entire control over the sphincter. On Saturday, being obliged to return home, I left her in charge of my friend, Dr. Thomas, who, under date of September 7th, writes as follows:—"Just eight days after the operation, Sarah was up walking about the house and yard—two days later she rode a short distance behind her mother on horseback—against orders, of course. Her general health is rapidly improving. She has entire control of the sphincter throughout the day. At night, when asleep, her old habit returns though in a gradually diminished degree—so much so, indeed, that I am confident it will soon disappear altogether."

Owing to being obliged to crush the stone before extraction, I am unable to make any very accurate statement as to its size. To my finger, when introduced into the bladder while the calculus was entire, it appeared of the volume of a hen's egg, and on putting the fragments, after removal, in juxtaposition with each other this was the opinion of the other medical gentlemen present.

I placed the stone in the hands of Dr. Haskins, of Clarksville, Tenn., for analysis, who has kindly furnished the annexed result, which, however, it is proper to remark, contains only the *prominent* constituent of each layer:

*Nucleus.*—Uric acid salts.

*Second Layer.*—Oxalate of lime.

*Third Layer.*—Phosphate of lime. Triple phosphate of magnesia and ammonia.

*Fourth and outer Layer.*—Oxalate of lime.—*Nashville Journal of Medicine and Surgery.*

